# Oxonium ammonio­(cyclo­prop­yl)methyl­enebis(hydrogenphospho­nate) monohydrate

**DOI:** 10.1107/S1600536808037094

**Published:** 2008-11-13

**Authors:** V. V. Bon, A. V. Dudko, A. N. Kozachkova, V. I. Pekhnyo

**Affiliations:** a V. I. Vernadskii Institute of General and Inorganic Chemistry, Kyiv 03680, Ukraine

## Abstract

The title compound, H_3_O^+^·C_4_H_10_NO_6_P_2_^−^·H_2_O, was obtained from the reaction of cyclo­propane­carbonitrile with PCl_3_, followed by dropwise addition of water. The asymmetric unit comprises an oxonium cation, a zwitterionic monoanion containing a positively charged ammonium group and two negatively charged phospho­nic acid residues and a water mol­ecule of crystallization. The hydroxonium cation and water mol­ecule are hydrogen bonded to the anion and further N—H⋯O and O—H⋯O bonds create a three-dimensional network.

## Related literature

Diphospho­nic acids are efficient drugs for the prevention of calcification and the inhibition bone resorption (Tromelin *et al.*, 1986 ▶, Matczak-Jon & Videnova-Adrabinska, 2005 ▶) and are used in the treatment of Pagets disease, osteoporosis and tumoral osteolysis (Szabo *et al.*, 2002 ▶). For bond-length data, see: Allen *et al.* (1987 ▶).
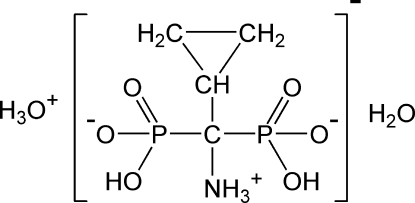


## Experimental

### 

#### Crystal data

H_3_O^+^·C_4_H_10_NO_6_P_2_^−^·H_2_O
*M* *_r_* = 267.11
Monoclinic, 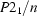

*a* = 12.5054 (8) Å
*b* = 5.6169 (4) Å
*c* = 14.3296 (8) Å
β = 94.973 (4)°
*V* = 1002.74 (11) Å^3^
*Z* = 4
Mo *K*α radiation
μ = 0.46 mm^−1^
*T* = 100 (2) K
0.56 × 0.07 × 0.06 mm

#### Data collection

Bruker SMART APEXII CCD diffractometer
Absorption correction: multi-scan (*SADABS*; Bruker, 2005 ▶) *T*_min_ = 0.782, *T*_max_ = 0.973
14938 measured reflections
2076 independent reflections
1411 reflections with *I* > 2σ(*I*)
*R*_int_ = 0.117

#### Refinement

*R*[*F* ^2^ > 2σ(*F* ^2^)] = 0.048
*wR*(*F* ^2^) = 0.111
*S* = 1.01
2076 reflections
166 parameters
6 restraints
H atoms treated by a mixture of independent and constrained refinement
Δρ_max_ = 0.64 e Å^−3^
Δρ_min_ = −0.48 e Å^−3^

### 

Data collection: *APEX2* (Bruker, 2005 ▶); cell refinement: *SAINT* (Bruker, 2005 ▶); data reduction: *SAINT*; program(s) used to solve structure: *SHELXTL* (Sheldrick, 2008 ▶); program(s) used to refine structure: *SHELXTL*; molecular graphics: *SHELXTL*; software used to prepare material for publication: *SHELXTL* and *PLATON* (Spek, 2003 ▶).

## Supplementary Material

Crystal structure: contains datablocks I, global. DOI: 10.1107/S1600536808037094/fj2162sup1.cif

Structure factors: contains datablocks I. DOI: 10.1107/S1600536808037094/fj2162Isup2.hkl

Additional supplementary materials:  crystallographic information; 3D view; checkCIF report

## Figures and Tables

**Table 1:** Hydrogen-bond geometry (Å, °)

*D*—H⋯*A*	*D*—H	H⋯*A*	*D*⋯*A*	*D*—H⋯*A*
N1—H1*A*⋯O4^i^	0.88 (4)	1.92 (4)	2.767 (4)	161 (3)
N1—H1*B*⋯O3^ii^	0.84 (3)	2.23 (3)	2.859 (4)	133 (3)
N1—H1*B*⋯O6^ii^	0.84 (3)	2.32 (3)	3.017 (4)	142 (3)
N1—H1*C*⋯O1^i^	0.86 (4)	2.05 (4)	2.846 (4)	154 (3)
O2—H2*O*⋯O1^iii^	0.78 (3)	1.75 (3)	2.521 (3)	178 (5)
O6—H6*O*⋯O3^ii^	0.81 (4)	1.70 (4)	2.508 (3)	171 (4)
O7—H71*O*⋯O4^iv^	0.81 (2)	1.79 (3)	2.600 (3)	171 (4)
O7—H72*O*⋯O8^ii^	0.82 (3)	1.76 (3)	2.555 (4)	164 (4)
O7—H73*O*⋯O5	1.09 (4)	1.35 (4)	2.441 (3)	175 (3)
O8—H82*O*⋯O2	0.82 (3)	2.12 (3)	2.871 (3)	153 (4)

Symmetry codes: (i) 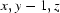; (ii) 
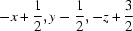
; (iii) 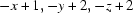; (iv) 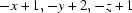.

**Fig. 1.**
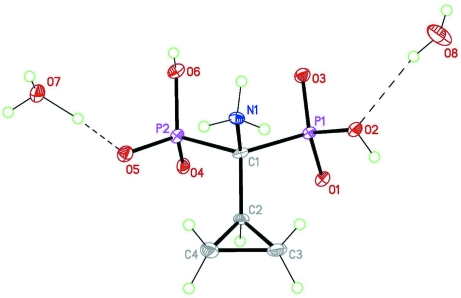
The asymmetric unit of title compound showing 50% probability displacement ellipsoids for the non-hydrogen atoms.

**Fig. 2.**
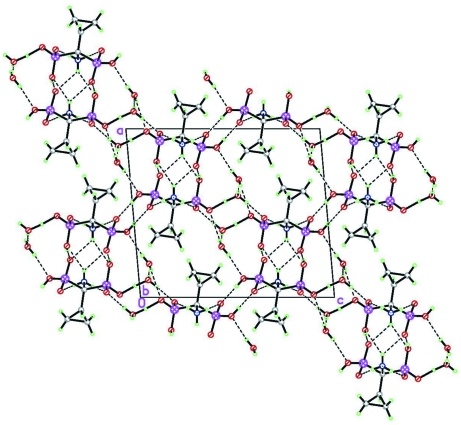
Crystal packing of title compound, projection along b axis. Dashed lines indicate hydrogen bonds.

## supplementary crystallographic information

### Comment

The organic diphosphonic acids are potentially very powerful chelating agents used in metal extractions and are tested by the pharmaceutical industry for use as efficient drugs preventing calcification and inhibiting bone resorption (Tromelin *et al.*, 1986, Matczak-Jon & Videnova-Adrabinska, 2005). Diphosphonic acids are used in the treatment of Paget disease, osteoporosis and tumoral osteolysis (Szabo *et al.*, 2002). The asymmetric unit of titled compound (Fig. 1) contains one molecule which exists as zwitterions with the proton transferred from one of the phosphonic group to the nitrogen atom. In the crystal structure of the compound the phosphorus atom displays a slightly distorted tetrahedral geometry provided by three oxygen atoms and one carbon atom. Bond lengths and angles have normal values (Allen *et al.*, 1987). The asymmetric unit contains one hydroxonium ion and one water molecule. The structure is stabilized by three-dimensional intramolecular O—H···O and N—H···O hydrogen bonds network (Table 1).

### Experimental

The preparation of oxonium ammonio(cyclopropyl)methylenebis(hydrogenphosphonate) hydrate was provided as follows. Dry hydrogen chloride at about 278 K was brought into contact with the surface of a mixture of cyclopropanecarbonitrile (73.7 ml, 1 mol) and PCl_3_ (87.4 ml, 1 mol) while stirring the mixture drop-wise addition of water (54 ml, 3 mol) was made in the molar ratio 1:1:3. After an a hour the solution becomes cloudy and sets. After cooling the product it was dissolved in water and separated by addition of acetone. The saturated solution was left at room temperature. Colourless crystals of the title compound were obtained after 1 week.

### Refinement

H atoms bonded to O and N atoms were located in a difference map. Other H atoms were positioned geometrically and refined using a riding model, with C—H = 0.99 Å for CH_2_ [*U*_iso_(H) = 1.2Ueq(N)] and C—H = 1.00 Å for CH [*U*_iso_(H) = 1.2Ueq(C)]. The strong H-bond between O5 and O7 was treated as an equilibrium between hydroxonium ion and water molecule. The position of the H atom was freely refined.

### Crystal data

**Table:**

H_3_O^+^·C_4_H_10_NO_6_P_2_^–^·H_2_O	*F*_000_ = 560
*M**_r_* = 267.11	*D*_x_ = 1.769 Mg m^−^^3^
Monoclinic, *P*2_1_/*n*	Melting point: 493 K
Hall symbol: -P 2yn	Mo *K*α radiation λ = 0.71073 Å
*a* = 12.5054 (8) Å	Cell parameters from 1935 reflections
*b* = 5.6169 (4) Å	θ = 2.3–26.4º
*c* = 14.3296 (8) Å	µ = 0.46 mm^−^^1^
β = 94.973 (4)º	*T* = 100 (2) K
*V* = 1002.74 (11) Å^3^	Needle, colourless
*Z* = 4	0.56 × 0.07 × 0.06 mm

### Data collection

**Table:**

Bruker SMART APEXII CCD diffractometer	2076 independent reflections
Radiation source: fine-focus sealed tube	1411 reflections with *I* > 2σ(*I*)
Monochromator: graphite	*R*_int_ = 0.117
*T* = 100(2) K	θ_max_ = 26.6º
φ and ω scans	θ_min_ = 2.1º
Absorption correction: multi-scan(SADABS; Bruker, 2005)	*h* = −15→15
*T*_min_ = 0.782, *T*_max_ = 0.973	*k* = −7→7
14938 measured reflections	*l* = −17→18

### Refinement

**Table:**

Refinement on *F*^2^	Secondary atom site location: difference Fourier map
Least-squares matrix: full	Hydrogen site location: inferred from neighbouring sites
*R*[*F*^2^ > 2σ(*F*^2^)] = 0.048	H atoms treated by a mixture of independent and constrained refinement
*wR*(*F*^2^) = 0.111	*w* = 1/[σ^2^(*F*_o_^2^) + (0.0546*P*)^2^] where *P* = (*F*_o_^2^ + 2*F*_c_^2^)/3
*S* = 1.01	(Δ/σ)_max_ < 0.001
2076 reflections	Δρ_max_ = 0.64 e Å^−^^3^
166 parameters	Δρ_min_ = −0.48 e Å^−^^3^
6 restraints	Extinction correction: none
Primary atom site location: structure-invariant direct methods	

### Special details

**Table:**

Geometry. All e.s.d.'s (except the e.s.d. in the dihedral angle between two l.s. planes) are estimated using the full covariance matrix. The cell e.s.d.'s are taken into account individually in the estimation of e.s.d.'s in distances, angles and torsion angles; correlations between e.s.d.'s in cell parameters are only used when they are defined by crystal symmetry. An approximate (isotropic) treatment of cell e.s.d.'s is used for estimating e.s.d.'s involving l.s. planes.
Refinement. Refinement of *F*^2^ against ALL reflections. The weighted *R*-factor *wR* and goodness of fit *S* are based on *F*^2^, conventional *R*-factors *R* are based on *F*, with *F* set to zero for negative *F*^2^. The threshold expression of *F*^2^ > σ(*F*^2^) is used only for calculating *R*-factors(gt) *etc*. and is not relevant to the choice of reflections for refinement. *R*-factors based on *F*^2^ are statistically about twice as large as those based on *F*, and *R*- factors based on ALL data will be even larger.

### Fractional atomic coordinates and isotropic or equivalent isotropic displacement parameters (Å^2^)

**Table:**

	*x*	*y*	*z*	*U*_iso_*/*U*_eq_	
P1	0.39124 (6)	1.00254 (15)	0.88098 (5)	0.0097 (2)	
P2	0.42829 (7)	0.97159 (15)	0.67003 (6)	0.0107 (2)	
C1	0.4541 (3)	0.8406 (5)	0.7872 (2)	0.0098 (7)	
C2	0.5754 (3)	0.8349 (6)	0.8120 (2)	0.0122 (7)	
H2A	0.6098	0.9951	0.8090	0.015*	
C3	0.6295 (3)	0.6770 (6)	0.8865 (2)	0.0161 (8)	
H3A	0.6897	0.7449	0.9275	0.019*	
H3B	0.5843	0.5615	0.9175	0.019*	
C4	0.6466 (3)	0.6358 (7)	0.7858 (2)	0.0186 (8)	
H4A	0.6120	0.4947	0.7546	0.022*	
H4B	0.7174	0.6780	0.7646	0.022*	
N1	0.4080 (2)	0.5913 (5)	0.7841 (2)	0.0098 (6)	
H1A	0.436 (3)	0.499 (6)	0.743 (3)	0.015*	
H1B	0.341 (2)	0.588 (6)	0.778 (2)	0.015*	
H1C	0.427 (3)	0.513 (6)	0.835 (3)	0.015*	
O1	0.46292 (18)	1.2100 (4)	0.91003 (15)	0.0121 (5)	
O2	0.38814 (18)	0.8132 (4)	0.95991 (15)	0.0121 (5)	
O3	0.27864 (18)	1.0669 (4)	0.84573 (15)	0.0138 (5)	
O4	0.46954 (18)	1.2202 (4)	0.67350 (15)	0.0130 (5)	
O5	0.48317 (18)	0.8051 (4)	0.60598 (15)	0.0137 (5)	
O6	0.30505 (19)	0.9707 (4)	0.64379 (16)	0.0133 (5)	
O7	0.3973 (2)	0.6970 (5)	0.45230 (18)	0.0263 (7)	
H2O	0.434 (3)	0.810 (7)	1.000 (2)	0.032*	
H6O	0.282 (3)	0.835 (8)	0.643 (3)	0.032*	
H71O	0.438 (3)	0.709 (7)	0.411 (2)	0.032*	
H72O	0.378 (3)	0.558 (5)	0.448 (3)	0.032*	
H73O	0.432 (3)	0.742 (7)	0.522 (3)	0.032*	
O8	0.1988 (2)	0.7952 (5)	1.0595 (2)	0.0285 (7)	
H81O	0.163 (3)	0.679 (6)	1.066 (3)	0.034*	
H82O	0.247 (3)	0.754 (7)	1.028 (3)	0.034*	

### Atomic displacement parameters (Å^2^)

**Table:**

	*U*^11^	*U*^22^	*U*^33^	*U*^12^	*U*^13^	*U*^23^
P1	0.0087 (5)	0.0105 (4)	0.0095 (4)	0.0000 (4)	−0.0012 (3)	−0.0002 (3)
P2	0.0116 (5)	0.0105 (5)	0.0096 (4)	−0.0001 (4)	−0.0008 (3)	−0.0003 (3)
C1	0.0068 (16)	0.0092 (17)	0.0126 (16)	−0.0009 (13)	−0.0029 (13)	0.0001 (13)
C2	0.0071 (17)	0.0149 (18)	0.0143 (17)	−0.0033 (14)	0.0004 (13)	−0.0038 (13)
C3	0.0088 (18)	0.020 (2)	0.0183 (18)	−0.0005 (15)	−0.0028 (14)	0.0006 (15)
C4	0.0114 (19)	0.024 (2)	0.0200 (19)	0.0026 (15)	0.0017 (15)	−0.0064 (15)
N1	0.0075 (15)	0.0090 (14)	0.0127 (14)	0.0008 (12)	0.0004 (12)	0.0001 (12)
O1	0.0133 (12)	0.0111 (12)	0.0112 (11)	0.0008 (10)	−0.0018 (9)	−0.0009 (9)
O2	0.0114 (13)	0.0148 (12)	0.0098 (11)	−0.0003 (10)	−0.0017 (9)	0.0006 (10)
O3	0.0115 (12)	0.0161 (13)	0.0136 (12)	0.0022 (10)	−0.0010 (9)	−0.0030 (9)
O4	0.0149 (13)	0.0148 (13)	0.0094 (11)	−0.0003 (10)	0.0011 (9)	0.0009 (9)
O5	0.0134 (12)	0.0140 (12)	0.0133 (12)	0.0033 (10)	−0.0011 (9)	−0.0012 (10)
O6	0.0135 (13)	0.0112 (12)	0.0145 (11)	−0.0009 (10)	−0.0028 (9)	−0.0004 (10)
O7	0.0203 (16)	0.0480 (18)	0.0108 (13)	−0.0117 (14)	0.0024 (11)	−0.0047 (14)
O8	0.0180 (16)	0.0335 (17)	0.0356 (17)	0.0026 (13)	0.0110 (13)	0.0120 (14)

### Geometric parameters (Å, °)

**Table:**

P1—O3	1.498 (2)	C3—H3B	0.9900
P1—O1	1.507 (2)	C4—H4A	0.9900
P1—O2	1.555 (2)	C4—H4B	0.9900
P1—C1	1.853 (3)	N1—H1A	0.88 (4)
P2—O4	1.488 (2)	N1—H1B	0.84 (3)
P2—O5	1.516 (2)	N1—H1C	0.86 (4)
P2—O6	1.554 (2)	O2—H2O	0.78 (3)
P2—C1	1.835 (3)	O5—H73O	1.35 (4)
C1—N1	1.514 (4)	O6—H6O	0.81 (4)
C1—C2	1.528 (4)	O7—H71O	0.81 (2)
C2—C4	1.498 (5)	O7—H72O	0.82 (3)
C2—C3	1.503 (5)	O7—H73O	1.09 (4)
C2—H2A	1.0000	O8—H81O	0.80 (3)
C3—C4	1.496 (5)	O8—H82O	0.82 (3)
C3—H3A	0.9900		
			
O3—P1—O1	115.19 (13)	C4—C3—H3A	117.8
O3—P1—O2	109.16 (13)	C2—C3—H3A	117.8
O1—P1—O2	112.32 (12)	C4—C3—H3B	117.8
O3—P1—C1	108.60 (13)	C2—C3—H3B	117.8
O1—P1—C1	107.49 (14)	H3A—C3—H3B	114.9
O2—P1—C1	103.34 (14)	C3—C4—C2	60.3 (2)
O4—P2—O5	115.18 (13)	C3—C4—H4A	117.7
O4—P2—O6	110.17 (13)	C2—C4—H4A	117.7
O5—P2—O6	110.06 (13)	C3—C4—H4B	117.7
O4—P2—C1	108.21 (13)	C2—C4—H4B	117.7
O5—P2—C1	104.70 (13)	H4A—C4—H4B	114.9
O6—P2—C1	108.16 (14)	C1—N1—H1A	113 (2)
N1—C1—C2	110.8 (3)	C1—N1—H1B	113 (3)
N1—C1—P2	107.9 (2)	H1A—N1—H1B	112 (3)
C2—C1—P2	108.3 (2)	C1—N1—H1C	111 (2)
N1—C1—P1	106.9 (2)	H1A—N1—H1C	100 (3)
C2—C1—P1	108.4 (2)	H1B—N1—H1C	106 (3)
P2—C1—P1	114.51 (17)	P1—O2—H2O	119 (3)
C4—C2—C3	59.8 (2)	P2—O5—H73O	119.6 (16)
C4—C2—C1	123.8 (3)	P2—O6—H6O	110 (3)
C3—C2—C1	123.7 (3)	H71O—O7—H72O	103 (4)
C4—C2—H2A	113.2	H71O—O7—H73O	115 (4)
C3—C2—H2A	113.2	H72O—O7—H73O	112 (4)
C1—C2—H2A	113.2	H81O—O8—H82O	106 (4)
C4—C3—C2	59.9 (2)		
			
O4—P2—C1—N1	177.8 (2)	O1—P1—C1—C2	39.6 (2)
O5—P2—C1—N1	−58.9 (2)	O2—P1—C1—C2	−79.4 (2)
O6—P2—C1—N1	58.4 (2)	O3—P1—C1—P2	43.8 (2)
O4—P2—C1—C2	−62.2 (2)	O1—P1—C1—P2	−81.46 (18)
O5—P2—C1—C2	61.1 (2)	O2—P1—C1—P2	159.60 (16)
O6—P2—C1—C2	178.4 (2)	N1—C1—C2—C4	31.4 (4)
O4—P2—C1—P1	58.9 (2)	P2—C1—C2—C4	−86.8 (3)
O5—P2—C1—P1	−177.81 (16)	P1—C1—C2—C4	148.4 (3)
O6—P2—C1—P1	−60.5 (2)	N1—C1—C2—C3	−42.3 (4)
O3—P1—C1—N1	−75.7 (2)	P2—C1—C2—C3	−160.5 (3)
O1—P1—C1—N1	159.06 (19)	P1—C1—C2—C3	74.7 (3)
O2—P1—C1—N1	40.1 (2)	C1—C2—C3—C4	112.7 (4)
O3—P1—C1—C2	164.8 (2)	C1—C2—C4—C3	−112.5 (4)

### Hydrogen-bond geometry (Å, °)

**Table:**

*D*—H···*A*	*D*—H	H···*A*	*D*···*A*	*D*—H···*A*
N1—H1A···O4^i^	0.88 (4)	1.92 (4)	2.767 (4)	161 (3)
N1—H1B···O3^ii^	0.84 (3)	2.23 (3)	2.859 (4)	133 (3)
N1—H1B···O6^ii^	0.84 (3)	2.32 (3)	3.017 (4)	142 (3)
N1—H1C···O1^i^	0.86 (4)	2.05 (4)	2.846 (4)	154 (3)
O2—H2O···O1^iii^	0.78 (3)	1.75 (3)	2.521 (3)	178 (5)
O7—H73O···O5	1.09 (4)	1.35 (4)	2.441 (3)	175 (3)
O6—H6O···O3^ii^	0.81 (4)	1.70 (4)	2.508 (3)	171 (4)
O7—H71O···O4^iv^	0.81 (2)	1.79 (3)	2.600 (3)	171 (4)
O7—H72O···O8^ii^	0.82 (3)	1.76 (3)	2.555 (4)	164 (4)
O8—H82O···O2	0.82 (3)	2.12 (3)	2.871 (3)	153 (4)

Symmetry codes: (i) *x*, *y*−1, *z*; (ii) −*x*+1/2, *y*−1/2, −*z*+3/2; (iii) −*x*+1, −*y*+2, −*z*+2; (iv) −*x*+1, −*y*+2, −*z*+1.

## References

[bb1] Allen, F. H., Kennard, O., Watson, D. G., Brammer, L., Orpen, A. G. & Taylor, R. (1987). *J. Chem. Soc. Perkin Trans. 2*, pp. S1–19.

[bb2] Bruker (2005). *APEX2*, *SAINT* and *SADABS* Bruker AXS Inc., Madison, Wisconsin, USA.

[bb3] Matczak-Jon, E. & Videnova-Adrabinska, V. (2005). *Coord. Chem. Rev.* **249**, 2458–2488.

[bb4] Sheldrick, G. M. (2008). *Acta Cryst.* A**64**, 112–122. 10.1107/S0108767307043930 18156677

[bb5] Spek, A. L. (2003). *J. Appl. Cryst.* **36**, 7–13.

[bb6] Szabo, Ch. M., Martin, M. B. & Oldfield, E. (2002). *J. Med. Chem.* **45**, 2894–2903. 10.1021/jm010279+ 12086477

[bb7] Tromelin, A., El Manouni, D. & Burgada, R. (1986). *Phosphorus Sulfur Relat. Elem.* **27**, 301–312.

